# Higher caries experience in individuals with neurofibromatosis 1: a matched case-control study

**DOI:** 10.1007/s00784-026-07066-5

**Published:** 2026-08-13

**Authors:** Eloá Borges Luna, Pâmella de Pinho Montovani, Flávia Lima, Analúcia Rampazzo Xavier, Rafaela Elvira Rozza-de-Menezes, Karin Soares Cunha

**Affiliations:** 1 Graduate Program in Pathology, School of Medicine, Universidade Federal Fluminense, Niterói, RJ Brazil; 2 Neurofibromatosis National Center (Centro Nacional de Neurofibromatose), Rio de Janeiro, RJ Brazil; 3 Department of Pathology, School of Medicine, Universidade Federal Fluminense, Niterói, RJ Brazil; 4 Graduate Program in Pathology, School of Medicine, Universidade Federal Fluminense, Rua Desembargador Athayde Parreira, 100, 5º andar, sala 2, Centro, Niterói, RJ 24033-900 Brazil

**Keywords:** Neurofibromatosis 1, Oral manifestations, Dental caries, DMFT, Saliva

## Abstract

**Objectives:**

This study evaluated dental caries experience in individuals with neurofibromatosis type 1 (NF1) compared with matched controls and investigated its associations with salivary parameters, behavioral factors, and socioeconomic characteristics.

**Materials and methods:**

In this case-control study, 42 individuals with NF1 and 42 controls matched for age and sex were included. Caries were diagnosed according to World Health Organization criteria, and the decayed, missing, and filled teeth (DMFT) index was used to assess caries experience. Oral hygiene was evaluated through the Simplified Oral Hygiene Index (OHI-S), tongue coating was quantified from standardized photographs, and questionnaires were used to assess cariogenic diet frequency and socioeconomic variables (educational level, housing status, and family income). Salivary assessments included unstimulated whole saliva flow rate (UWSFR), chewing-stimulated whole saliva flow rate (CSWSFR), pH, buffering capacity, viscosity, and levels of calcium, phosphorus, potassium, magnesium, amylase, and lipase.

**Results:**

Participants with NF1 showed higher caries experience than matched controls (DMFT index: 18.5 ± 8.6 vs. 12.6 ± 7.4; *p* = 0.001). Hyposalivation (UWSFR: 57.1% vs. 2.4%; CSWSFR: 40.5% vs. 0%) and high salivary viscosity (71.4% vs. 11.9%) were significantly more frequent in NF1 (all *p* < 0.001). While bivariate analysis within the NF1 group showed no direct association between DMFT and salivary flow, post hoc power analysis indicated limited statistical power (20.4%) for this comparison. A significant positive correlation was found between DMFT and tongue coating index (ρ = 0.312; *p* = 0.044). In multivariable regression, NF1 was independently associated with higher DMFT compared to matched controls (*p* < 0.001). Additionally, lower family income (*p* < 0.001) and poorer oral hygiene (*p* = 0.016) were significantly associated with higher DMFT. A significant interaction between group and cariogenic diet (*p* < 0.001) was also observed, indicating that the association between dietary habits and DMFT differed between groups.

**Conclusions:**

Individuals with NF1 present significantly higher DMFT compared to matched controls. Our findings suggest that this is a multifactorial process, characterized by an independent association between NF1 status and DMFT, even after adjusting for salivary flow, oral hygiene, socioeconomic status, and dietary habits. Notably, the high prevalence of hyposalivation and altered salivary viscosity—combined with the significant positive correlation between tongue coating and DMFT—highlight a complex and compromised oral environment in NF1.

**Clinical relevance:**

These findings support that individuals with NF1 represent a high-risk group for dental caries and significant salivary impairment. This dual vulnerability necessitates specialized preventive protocols, including intensive biofilm control and regular dental monitoring, to reduce the high cumulative caries experience and its impact on oral health in this population.

## Introduction

 Neurofibromatosis 1 (NF1) is an autosomal dominant disorder with a prevalence of approximately 1 in 3,000 individuals worldwide [1]. It results from pathogenic variants in the *NF1* gene, which encodes neurofibromin, a key negative regulator of Ras signaling, promoting the conversion of active Ras-GTP to inactive Ras-GDP through its GTPase-activating protein (GAP) function [2, 3]. Neurofibromin is expressed across numerous cell types, playing a central role in maintaining cellular homeostasis and regulating proliferation [4]. NF1 is associated with both benign and malignant tumors, as well as non-tumoral alterations [5]. 

The most common manifestations of NF1 include multiple neurofibromas, café-au-lait spots, axillary and inguinal freckling, Lisch nodules, choroidal abnormalities, and skeletal alterations [5]. Oral manifestations occur in over 90% of individuals and include enlarged fungiform papillae, intraoral neurofibromas, gingival enlargement, malocclusion, retained teeth, and structural bone alterations such as enlargement of the mental and mandibular foramina and mandibular canal [6–9]. 

From a mechanistic perspective, Ras signaling is essential for the morphogenesis and differentiation of ectoderm-derived tissues, particularly neural crest–derived structures [10]. It plays a key role in odontogenesis, regulating enamel and dentin formation and playing a central role in salivary gland development, homeostasis, and regeneration [11–13]. Supporting this biological link, our group previously demonstrated neurofibromin expression in major and minor salivary glands in individuals without NF1, indicating its role in glandular homeostasis [14]. Reduced neurofibromin function in NF1 may therefore result in structural or functional impairments in these tissues, potentially contributing to oral alterations such as hyposalivation and increased dental caries susceptibility. Consistently, our group has also shown high prevalence of hyposalivation in individuals with NF1 compared to matched controls [15]. 

Saliva plays a central role in oral homeostasis [16]. Reduced salivary flow is associated with increased caries risk due to impaired mechanical clearance, buffering capacity, and remineralization [16–18]. Beyond flow rate, saliva contains key components involved in oral defense [16, 17]. Calcium and phosphate are essential for enamel remineralization and mineral balance, while magnesium may influence dental mineralization [16–18]. Other electrolytes, such as potassium, contribute to ionic balance and physicochemical properties [16–18]. Salivary pH and buffering capacity are critical for acid neutralization and prevention of demineralization [16]. Enzymes such as amylase and lipase contribute to the biochemical composition of saliva and may influence the oral environment [16, 19]. Additionally, rheological properties, including viscosity, play a role in lubrication and oral clearance [16]. 

Clinical investigations into the caries experience in NF1 have yielded heterogeneous findings. While some studies report higher dental caries experience in individuals with NF1, others show no significant differences or even lower experience compared to controls [20–26]. These discrepancies likely reflect methodological differences, including variations in age groups, diagnostic criteria, and challenges in separating intrinsic disease effects from environmental and socioeconomic factors.

Based on this rationale, we hypothesized that individuals with NF1 have higher caries experience associated with reduced salivary flow compared to controls. To address gaps in the literature, we used a matched case-control design to evaluate caries experience and salivary function in NF1, as well as their associations with salivary parameters, behavioral factors, and socioeconomic characteristics.

## Materials and methods

This study was approved by the Ethics Committee of Antônio Pedro University Hospital (HUAP), Universidade Federal Fluminense (UFF), Brazil (#1.645.631, 2016). Data were collected between 2017 and 2022 at the Oral Diagnosis Clinic and the Clinical Analyses and Pathological Anatomy Unit of HUAP, as well as at the School of Dentistry, UFF. Written informed consent was obtained from all participants.

### Sample

The study included a convenience sample of individuals with NF1 and matched controls. The NF1 diagnosis was based on the revised National Institutes of Health (NIH) criteria [5]. To ensure comparability and minimize the effect of cumulative caries experience over time, a 1:1 matching process was performed, pairing each participant with a control of the same age and sex. This ensured equivalent exposure time to caries risk in both groups.

The present study was conducted using a partially overlapping study population from a parallel investigation into periodontal disease in NF1 [27]. 

### Methods

All clinical examinations and data collection were performed by a single experienced Oral Medicine specialist using standardized diagnostic criteria, thereby avoiding inter-examiner variability.

Age and sex were recorded for all participants during the initial interview. Individuals were grouped into three life stages—adolescents (10–19 years), adults (20–59 years), and elderly (60 years or older).

#### Assessment of caries experience

Dental caries experience was assessed using the DMFT index (“D” = decayed, “M” = missing, “F” = filled teeth) [28, 29]. Each decayed, missing, or filled tooth was assigned a score of 1 in an odontogram. Caries diagnosis followed World Health Organization (WHO) criteria, including visible cavities on smooth surfaces, grooves, or the floor/soft walls of tooth enamel [29]. A probe tip could enter the cavity of the tooth’s proximal surfaces to be recorded as decayed; in doubtful cases, teeth were considered sound [29]. Restored teeth with recurring caries were also classified as decayed. Suspicious areas or white spots were not included [29]. Missing teeth were recorded as “M,” except for those where the participant explicitly reported extraction due to trauma or orthodontic indications. Teeth with intact restorations and no caries were classified as filled. The DMFT score, ranging from 0 to 28 (excluding third molars), was calculated as the sum of its components. Edentulous individuals were assigned a DMFT score of 28.

The level of caries experience in permanent dentition was categorized based on WHO severity criteria: [28, 29] very low (< 5.0), low (5.0–8.9), moderate (9.0–13.9) and high (> 13.9). For statistical analysis, caries experience was grouped into non-high (very low, low, and moderate) and high categories.

#### Evaluation of salivary parameters

##### Measurement of Salivary Flow Rates

Sialometry was performed in the morning (9:30–10:30 a.m.) [30]. Unstimulated whole saliva flow rate (UWSFR) was measured by passive drooling for 5 min after an initial swallow. For chewing-stimulated whole saliva flow rate (CSWSFR), participants chewed a mechanical sialogogue for 1 min, swallowed, and then continued chewing for 5 min while saliva was collected. Saliva volume from both procedures was measured separately using a disposable 10 mL syringe, considering only the liquid component, and flow rate (mL/min) was calculated. Only the liquid component (excluding foam) was measured. Values were classified according to a previous study [15]. Hyposalivation was defined as < 0.3 mL/min for UWSFR and < 0.7 mL/min for CSWSFR.

##### Evaluation of Salivary Viscosity

After measuring salivary flow, viscosity was assessed using the visual thread method. Saliva was aspirated from an Eppendorf tube with a syringe to form a thread; a thick, sticky thread indicated high viscosity, whereas a clear, fluid thread indicated normal viscosity.

##### Measurement of Salivary pH and Buffering Capacity

The pH and buffering capacity of saliva were evaluated using pH indicator strips (MColorpHast™; Merck Chemical). Saliva was collected both at rest and under stimulation. Following collection, a refrigerated centrifuge (24 × 2 mL/rcf maximum 21,380 × g) was used to spin the samples at 10,000 rpm for 15 min at 4 °C. Normal salivary pH was considered to be within the range of 6.5–7.5 [31].

Salivary buffering capacity was assessed using stimulated saliva. Participants rinsed with a citric acid solution for 30 s and discarded the saliva; pH was then measured and expected to be acidic (pH < 7.0). After rinsing with distilled water, a 10-minute interval was observed before a new saliva sample was collected and pH remeasured. Buffering capacity was considered adequate if pH returned to baseline values.

##### Analysis of Inorganic (Calcium, Phosphorus, Potassium, Magnesium) and Organic (Enzymes) Salivary Components

For the analysis of inorganic salivary components, unstimulated and stimulated saliva samples were centrifuged separately at 10,000 rpm for 15 min at 4 °C (SL-5GR, Spinlab, Brazil). The supernatant was collected, aliquoted into 2 mL cryogenic tubes, and analyzed. Calcium (mg/dL), potassium (mEq/L), and magnesium (mg/dL) were measured using an ion-selective electrode analyzer (Cobas b 123^®^, Roche Diagnostics, USA). Phosphorus (mg/dL), as well as salivary amylase and lipase (U/mg protein), were determined using an automated spectrophotometer (Selectra Flexor E, ElitechGroup, USA) with Kovalent reagents, according to the manufacturer’s instructions. Absolute values were used for statistical analysis.

#### Assessment of oral hygiene

To evaluate oral hygiene, the Simplified Oral Hygiene Index (OHI-S) was used [32]. After performing relative isolation of the teeth with cotton rolls, dental biofilm was disclosed using a dye solution (Eviplac, Biodynamic, Brazil) to facilitate visualization of the Debris Index (DI-S). Dental calculus was assessed visually according to the Calculus Index (CI-S) criteria. Each component was scored separately from 0 to 3 according to the extent of surface coverage (0 = absent; 1 = up to 1/3 of the dental surface; 2 = up to 2/3 of the dental surface; 3 = more than 2/3 of the dental surface) [32]. 

The result was calculated by summing DI-S and CI-S, then dividing by the total number of buccal and lingual surfaces examined. The index was classified as follows: good (0 to 1), regular (1.1 to 2.0), and poor (2.1 to 3.0) [32]. 

#### Assessment of tongue coating

Tongue coating was assessed from standardized photographs of the dorsal tongue surface, obtained under consistent lighting and positioning conditions. The images were analyzed by dividing the tongue surface into nine regions, each scored from 0 to 2 (0 = no coating, 1 = thin coating, 2 = thick coating) [33]. The values for each part were summed, divided by 18, and then multiplied by 100 to calculate the percentage of the tongue coating index. Results were classified as light coating (≤ 50% of the tongue surface covered) or severe coating (> 50% of the tongue surface covered).

#### Assessment of cariogenic diet

Cariogenic diet was initially assessed using a food frequency questionnaire focusing on sugar intake [34, 35]. The frequency of consumption of sugar-containing beverages, cookies, candies, chewing gum, and soft drinks was recorded over a 7-day period. Weekly consumption was categorized as > 5 days (high frequency), 3–5 days (intermediate frequency), and < 3 days (low frequency).

#### Socioeconomic status

Socioeconomic status was assessed by educational level (incomplete or complete secondary education), housing status (homeowner or renter), and family income (in number of minimum wages) [36]. 

## Statistical analyses

Descriptive analyses and paired inferential tests were performed using SPSS (version 20.0; IBM^®^, USA). Comparisons between NF1 and control groups were conducted using paired tests (Wilcoxon signed-rank for continuous variables and McNemar’s test for categorical variables). Within-group analyses in the NF1 group explored associations between DMFT and clinical, salivary, behavioral, and sociodemographic variables.

Normality was assessed using the Shapiro–Wilk test. Given non-normal distributions and small subgroup sizes, non-parametric methods were applied. Comparisons across subgroups were performed using the Mann–Whitney or Kruskal–Wallis tests, and correlations were assessed using Spearman’s rank correlation. All tests were two-tailed, with *p* ≤ 0.05 considered statistically significant.

An exploratory post hoc power analysis was conducted for selected non-significant variables showing relevant descriptive differences, using G*Power (version 3.1.9.6; University of Düsseldorf, Germany) with α = 0.05.

To evaluate factors associated with DMFT and the independent effect of NF1, a multivariable generalized linear mixed model (GLMM) was fitted using Jamovi (version 2.6.45.0) with the GAMLj module. A Poisson distribution with a log link was assumed, with DMFT as the dependent variable and group (NF1 vs. control) as the main predictor, adjusted for family income, oral hygiene (OHI-S), UWSFR, and cariogenic diet. Variables were selected based on clinical relevance and prior evidence, and interaction terms were tested. The matched design was accounted for by including pair ID as a random effect. Results are presented as exponentiated coefficients [Exp(B)] with 95% confidence intervals. Overdispersion was assessed using the Pearson χ²/degrees of freedom ratio.

### Participant demographics

The study included 84 participants: 42 individuals with NF1 and 42 matched controls. Groups were matched for age and sex, comprising 32 women and 10 men each, aged 18–77 years (mean 39.7 ± 15.3 years). Both groups included 7 adolescents, 32 adults, and 3 elderly individuals.

### Increased DMFT in NF1 individuals compared to matched controls

The NF1 group showed significantly higher DMFT than controls (18.5 vs. 12.6; *p* = 0.001, Wilcoxon signed-rank test; Table 1). Age-stratified analysis revealed higher DMFT in adults with NF1 compared to matched controls (20.3 vs. 13.0; *p* < 0.001, Wilcoxon signed-rank test), while no significant difference was observed among adolescents, with wide confidence intervals. The elderly subgroup (*n* = 3) was not analyzed inferentially due to the small sample size and is presented descriptively in Table 1.

**Table 1:** Bivariate comparison of clinical, salivary, biochemical and sociodemographic variables between NF1 individuals and matched controls

Variables	Pairs (*n*)	NF1	Matched controls	*p*-value
Dental caries experience (DMFT)	42	**mean ± sd**	**mean ± sd**	
Total DMFT		18.5 (± 8.6)	12.6 (± 7.4)	**0.001¹**
Decayed		2.1 (± 3.7)	1.2 (± 2.0)	
Missing		5.2 (± 8.6)	2.3 (± 3.8)	
Filled		11.1 (± 6.6)	8.9 (± 4.9)	
DMFT stratified by sex		**mean ± sd**	**mean ± sd**	
Female	32	19.2 (± 9.0)	13.3 (± 7.1)	**0.005¹**
Male	10	16.5 (± 6.9)	10.0 (± 7.9)	**0.050¹**
DMFT stratified by age group		**mean ± sd**	**mean ± sd**	
Adolescents	7	7.9 (± 8.8)	6.3 (± 4.7)	0.734¹
Adults	32	20.3 (± 7.0)	13.0 (± 6.9)	**< 0.001¹**
Elderly	3	25.7 (± 4.0)	22.7 (± 6.1)	—*
WHO caries severity	42	**n (%)**	**n (%)**	
Non-high		11 (26.2%)	22 (52.4%)	**0.08²**
High experience		31 (73.8%)	20 (47.6%)	
Salivary flow rate	42			
Unstimulated flow (UWSF) (mL/min) (mean ± sd)		0.29 (± 0.2)	0.67 (± 0.3)	**< 0.001¹**
Stimulated flow (CSWSFR) (mL/min) (mean ± sd)		0.79 (± 0.3)	1.17 (± 0.3)	**< 0.001¹**
Hyposalivation (UWSFR) n (%)		24 (57.1%)	1 (2.4%)	**< 0.001** ^**2**^
Hyposalivation (CSWSFR) n (%)		17 (40.5%)	0 (0%)	**< 0.001** ^**2**^
Salivary viscosity	42	**n (%)**	**n (%)**	
High		30 (71.4%)	5 (11.9%)	**< 0.001** ^**2**^
Salivary pH		**mean ± sd**	**mean ± sd**	
Unstimulated saliva	38**	6.82 (± 0.56)	6.79 (± 0.58)	0.773^1^
Stimulated saliva	42	7.57 (± 0.5)	7.57 (± 0.55)	1.000^1^
Salivary buffering capacity	34**	**n (%)**	**n (%)**	
Adequate		34 (100%)	34 (100%)	NA
Salivary biochemical & enzymatic (unstimulated)		**mean ± sd**	**mean ± sd**	
Calcium (mg/dL)	38**	3.96 (± 2.25)	4.13 (± 1.99)	0.879¹
Phosphorus (mg/dL)	38**	10.64 (± 6.29)	11.65 (± 5.49)	0.542¹
Potassium (mEq/L)	38**	12.04 (± 7.50)	11.46 (± 3.48)	0.794¹
Magnesium (mg/dL)	38**	0.91 (± 1.19)	1.52 (± 1.95)	0.112¹
Amylase (U/mg protein)	19**	5141.9 (± 7266.3)	10859.6 (± 14821.7)	0.104¹
Lipase (U/mg protein)	19**	0.92 (± 1.86)	0.39 (± 0.77)	0.286¹
Salivary biochemical & enzymatic (stimulated)		**mean ± sd**	**mean ± sd**	
Calcium (mg/dL)	42	4.03 (± 2.33)	4.04 (± 1.70)	0.984¹
Phosphorus (mg/dL)	42	10.10 (± 6.0)	11.36 (± 4.33)	0.255¹
Potassium (mEq/L)	42	13.20 (± 8.13)	11.65 (± 4.04)	0.418¹
Magnesium (mg/dL)	42	0.94 (± 1.14)	0.86 (± 0.95)	0.697¹
Amylase (U/mg protein)	20	5277.2 (± 7889.6)	16305.2 (± 27198.5)	0.105¹
Lipase (U/mg protein)	20	0.52 (± 0.82)	19.14 (± 85.25)	0.178¹
Oral hygiene index (OHI-S)	34			
OHI-S score (mean ± sd)		1.32 (± 0.6)	1.26 (± 0.6)	0.694¹
Good n (%)		25 (73.5%)	32 (76.2%)	1.000²
Regular n (%)		8 (23.5%)	9 (21.4%)	
Poor n (%)		1 (2.9%)	1 (2.4%)	
Tongue coating	42			
Tongue coating index (%) (mean ± sd)		44.5 (± 21.7)	33.7 (± 22.6)	**0.030¹**
Severe coating (> 50%) n (%)		18 (42.9%)	12 (28.6%)	0.210²
Cariogenic diet frequency	42	**n (%)**	**n (%)**	
Low frequency		10 (23.8%)	11 (26.2%)	0.782¹
Intermediate frequency		7 (16.7%)	9 (21.4%)	
High frequency		25 (59.5%)	22 (52.4%)	
Educational level	42	**n (%)**	**n (%)**	
Incomplete secondary education		5 (11.9%)	1 (2.4%)	0.219²
Housing status	42	**n (%)**	**n (%)**	
Renter		8 (19%)	19 (45.2%)	**0.007²**
Family income (minimum wages) (mean ± sd)	42	4.0 (± 3.6)	4.5 (± 2.4)	0.141¹

Abbreviations: *NF1* neurofibromatosis type 1, *DMFT* decayed, missing, and filled teeth, *OHI-S* Simplified Oral Hygiene Index, —*, not tested due to small sample size (*n* = 3), —** no individuals in this category in the control group, *NA* not applicable (no variability between groups), ** the number of observations (n) varies across variables due to technical limitations related to saliva collection and laboratory processing

Some samples did not meet the minimum volume required for all analyses or presented incomplete measurements after centrifugation and handling

Statistical tests: ^1^ Wilcoxon signed-rank test; ^2^ McNemar’s test

Consistent with these findings, a statistically significant positive correlation was observed between age and DMFT in the NF1 group (Spearman’s ρ = 0.516, *p* < 0.001; Table 2). Caries experience was classified as high in 73.8% of the NF1 group and 47.6% of matched controls; however, no statistically significant difference was observed for this distribution (Table 1), with a post hoc power of 61.7%.

**Table 2:** Bivariate analysis of DMFT according to clinical, salivary, behavioral and sociodemographic variables in individuals with NF1

Variables	NF1			*p*-value
	*n*/*N*	DMFT (mean±sd)	Spearman’s rho	
Sex				
Female	32/42	19.2 (± 9)	NA	0.236^1^
Male	10/42	16.5 (± 6.9)	NA	
AGE				
All individuals	42	NA	**0.516** (rho)	**< 0.001** ^2^
Adolescents	7/42	7.9 (± 8.8)	NA	**0.004** ^3^
Adults	32/42	20.3 (± 7.0)	NA	
Elderly	3/42	25.7 (± 4.0)	NA	
Salivary flow rate				
Unstimulated whole saliva flow rate (UWSFR)				
Flow rate	42	NA	−0.178 (rho)	0.260²
Hyposalivation	28/42	18.8 (± 8.7)	NA	0.797^1^
Normal salivary flow	14/42	18.1 (± 8.7)	NA	
Chewing-stimulated whole saliva flow rate (CSWSFR)				
Flow rate	42	NA	−0.138 (rho)	0.384²
Hyposalivation	17/42	20.1 (± 8.0)	NA	0.450^1^
Normal salivary flow	25/42	17.6 (± 5.1)	NA	
Salivary viscosity	n/N			
Normal	12/42	16.8 (± 7.1)	NA	0.224^1^
High viscosity	30/42	19.3 (± 9.2)	NA	
Salivary buffering capacity				
Adequate	34/34	18.8 (± 8.2)	NA	—
Inadequate	0/34	—	NA	
Simplified oral hygiene index (OHI-S)				
OHI-S	34	NA	0.236 (rho)	0.201^2^
Good	25/34	17.0 (± 8)	NA	0.260^3^
Regular	8/34	20.4 (± 10.5)	NA	
Poor	1/34	2.0	NA	
Tongue coating index				
Tongue coating index (%)	42/42	NA	0.312 (rho)	**0.044**²
Light coating (≤ 50%)	24/42	16.9 (± 8.7)	NA	0.121^1^
Severe coating (> 50%)	18/42	20.8 (± 8.2)	NA	
Cariogenic diet frequency				
High frequency	25/42	20.5 (± 8.2)	NA	0.168^3^
Intermediate frequency	7/42	14.0 (± 8.1)	NA	
Low frequency	10/42	17.0 (± 9.2)	NA	
Educational level				
Incomplete secondary education	5/42	22.2 (± 5.8)	NA	0.503^1^
Complete secondary education	37/42	18.1 (± 8.9)	NA	
Housing status				
Homeowner	34/42	17.6 (± 8.8)	NA	0.188^1^
Renter	8/42	22.6 (± 6.7)	NA	
Family income				
Number of minimum wages	42/42	NA	−0.211 (rho)	0.181^2^

Abbreviations: *NF1* neurofibromatosis type 1, *DMFT* decayed, missing, and filled teeth

Statistical tests: ^1^Mann-Whitney test; ^2^Spearman’s correlation test; ^3^Kruskal-Wallis test

### Salivary parameters

#### Reduced salivary flow and increased hyposalivation in NF1 individuals

Individuals with NF1 showed significantly lower salivary flow rates than matched controls under both unstimulated and stimulated conditions (*p* < 0.001, Wilcoxon signed-rank test; Table 1). Accordingly, hyposalivation was more frequent in the NF1 group for both unstimulated (57.1% vs. 2.4%) and stimulated flow (40.5% vs. 0.0%) (*p* < 0.001, McNemar’s test; Table 1).

Despite the high prevalence of hyposalivation in the NF1 group, no significant association was observed between UWSFR or CSWSFR and DMFT, either as continuous variables or categorically (hyposalivation vs. normal flow; Table 2). The post hoc power for the UWSFR correlation was 20.4%, indicating limited ability to detect associations with the current sample size.

#### Increased salivary viscosity in NF1 individuals

High salivary viscosity was significantly more frequent in NF1 than in matched controls (71.4% vs. 11.9%; *p* < 0.001, McNemar’s test; Table 1). However, no significant association was observed between salivary viscosity and DMFT in the NF1 group (Table 2).

#### No differences in salivary pH and buffering capacity between NF1 and matched controls

Salivary pH did not differ between NF1 and control groups under unstimulated or stimulated conditions (Table 1), and all participants showed adequate buffering capacity. Within the NF1 group, no significant correlation was observed between DMFT and salivary pH (Table 3). Buffering capacity was not analyzed in relation to DMFT due to lack of variability, as all participants in both groups showed adequate results.

**Table 3:** Bivariate correlation between DMFT and salivary pH, and inorganic and organic components in individuals with NF1

Variable	Saliva Condition	*n*	Spearman’s ρ	*p*-value^1^
Calcium	Unstimulated saliva	39*	−0.293	0.071
Calcium	Stimulated saliva	42	−0.259	0.097
Phosphorus	Unstimulated saliva	39*	−0.007	0.966
Phosphorus	Stimulated saliva	42	−0.145	0.361
Potassium	Unstimulated saliva	39*	0.047	0.778
Potassium	Stimulated saliva	42	0.002	0.991
Magnesium	Unstimulated saliva	39*	0.136	0.410
Magnesium	Stimulated saliva	42	−0.019	0.903
Salivary amylase	Unstimulated saliva	20*	−0.226	0.337
Salivary amylase	Stimulated saliva	21*	−0.234	0.308
Salivary lipase	Unstimulated saliva	20*	0.103	0.665
Salivary lipase	Stimulated saliva	21*	0.104	0.655
Salivary pH	Unstimulated saliva	39*	−0.281	0.083
Salivary pH	Stimulated saliva	42	0.024	0.878

Abbreviations: *NF1* neurofibromatosis type 1, *DMFT* decayed, missing, and filled teeth

*The number of observations (n) varies across variables due to technical limitations related to saliva collection and laboratory processing

Some samples did not meet the minimum volume required for all analyses or presented incomplete measurements after centrifugation and handling

Statistical test: ^1^Spearman's correlation

#### No differences in inorganic and organic salivary components between NF1 and matched controls

No significant differences were observed between the NF1 and matched control groups regarding inorganic components or amylase and lipase levels under unstimulated or stimulated conditions (Table 1).

Within the NF1 group, no significant correlations were detected between DMFT and levels of phosphorus, potassium, magnesium, or with salivary enzymes (Table 3).

### No differences in oral hygiene between NF1 individuals and matched controls

Eight NF1 participants were excluded from the OHI-S analysis (four edentulous and four using orthodontic appliances), resulting in a final sample of 34. No significant difference was observed between groups in OHI-S scores (Table 1). When categorized, 73.5% of NF1 individuals showed good, 23.5% regular, and 2.9% poor oral hygiene, with a similar distribution in controls (Table 1). Within the NF1 group, no significant association was observed between OHI-S and DMFT (Table 2).

### Increased tongue coating in NF1 individuals

The tongue coating index was higher in individuals with NF1 than in controls (*p* = 0.030, Wilcoxon signed-rank test; Table 1). When categorized, severe tongue coating (> 50%) was more frequent in the NF1 group (42.9% vs. 28.6%), although this difference was not significant (Table 1). Within the NF1 group, a significant positive correlation was observed between tongue coating index and DMFT (Spearman’s ρ = 0.312, *p* = 0.044; Table 2).

### Cariogenic diet

A high-frequency cariogenic diet was observed in 59.5% of individuals with NF1 and 52.4% of controls, with no significant difference between groups (Table 1). Within the NF1 group, no significant association was found between cariogenic diet and DMFT (Table 2).

### Socioeconomic characteristics

Regarding education, 11.9% of individuals with NF1 had incomplete secondary education versus 2.4% of controls, with no significant difference between groups (Table 1). In contrast, a lower proportion of NF1 individuals were renters (19.0% vs. 45.2%), representing a significant difference (*p* = 0.007, McNemar’s test; Table 1). Mean family income was similar between groups (4.0 vs. 4.5 minimum wages; Table 1).

Within the NF1 group, no significant associations were observed between DMFT and educational level, housing status, or family income (Table 2).

### Multivariable analysis

A mixed-effects Poisson regression model with a random intercept for matched pairs was used to evaluate factors associated with DMFT (Table 4). Mild-to-moderate overdispersion was observed (χ²/df = 1.60). The Poisson model was retained, as the negative binomial alternative resulted in a singular fit and unstable estimates. The final model showed consistent estimates and a high conditional R² (0.865).

**Table 4:** Multivariable mixed-effects Poisson regression model for DMFT values

Predictor	Estimate (B)	SE	Exp(B) (95% CI)	z	*p*-value
(Intercept)	2.461	0.068	11.716 (10.263–13.374)	36.43	< 0.001
Group (Control - NF1)	−0.518	0.115	0.596 (0.476–0.747)	−4.50	< 0.001
Family income (per unit increase)	−0.053	0.016	0.948 (0.919–0.978)	−3.33	< 0.001
OHI-S	0.188	0.078	1.207 (1.036–1.406)	2.41	0.016
UWSFR	−0.284	0.177	0.753 (0.532–1.066)	−1.60	0.110
Cariogenic diet (Intermediate vs. high)	−0.977	0.135	0.376 (0.289–0.491)	−7.22	< 0.001
Cariogenic diet (Low vs. high)	−0.124	0.090	0.883 (0.740–1.054)	−1.38	0.169
Group × Diet (Control-NF1 x Intermediate-high)	−1.075	0.272	0.341 (0.200–0.582)	−3.95	< 0.001
Group × Diet (Control-NF1 x low-high)	−0.229	0.218	0.795 (0.519–1.218)	−1.05	0.292

Abbreviations: *DMFT* decayed, missing, and filled teeth, *OHI-S* Simplified Oral Hygiene Index, *UWSFR* unstimulated whole salivary flow rate

Model: Mixed-effects Poisson regression with random intercept for matched pairs

Reference categories: NF1 group; low cariogenic diet

Note: All covariates were centered at their mean values. Interaction terms represent the joint effect of group and cariogenic diet on DMFT; Number of Obs: 76

In the adjusted model, NF1 was independently associated with higher DMFT, as controls showed lower values (Exp(B) = 0.596, 95% CI: 0.476–0.747; *p* < 0.001). Family income showed a significant inverse association with DMFT (Exp(B) = 0.948, 95% CI: 0.919–0.978; *p* < 0.001), indicating that lower income levels were associated with higher DMFT after adjustment. Poorer oral hygiene was also a significant predictor, with higher OHI-S scores associated with increased DMFT (Exp(B) = 1.207, 95% CI: 1.036–1.406; *p* = 0.016).

A significant interaction between group and cariogenic diet was observed (*p* < 0.001), suggesting that the impact of dietary habits on caries experience differs between NF1 individuals and matched controls. While an intermediate frequency of cariogenic diet (vs. high) showed an overall protective effect in the model (Exp(B) = 0.376, 95% CI: 0.289–0.491; *p* < 0.001), the interaction terms reveal that this association was significantly modulated by the group (Control × Intermediate vs. High: Exp(B) = 0.341, 95% CI: 0.200–0.582; *p* < 0.001). This indicates that the reduction in DMFT expected from dietary improvement is significantly more pronounced in the control group than in the *NF1* population.

## Discussion

This study provides a multidimensional assessment of dental caries experience in individuals with NF1, integrating salivary, behavioral, and socioeconomic factors. Individuals with NF1 showed significantly higher DMFT than matched controls.

Previous studies on dental caries in NF1 have reported heterogeneous findings, likely reflecting methodological differences. Tucker et al. [20], in a mixed-age population, suggested increased caries experience; however, the retrospective design and reliance on self-reported data limit these findings. Friedrich et al. [22], in a study including adolescents and adults, reported higher DMFT in NF1 compared to controls, primarily driven by missing teeth rather than active carious lesions. However, their reliance solely on panoramic radiographs may have underestimated early lesions. More recently, a small case–control study (*n* = 11) reported higher DMFT in individuals over 20 years of age, with no significant differences in younger participants [26].

Other studies in children and adolescents have reported no significant differences in caries experience between individuals with NF1 and controls [24, 25]. Visnapuu et al. [21], in a study including a mixed-age population, reported lower DMFT in individuals with NF1 compared with the general population, particularly in younger age groups, with differences diminishing with age. Similarly, a recent study found fewer decayed teeth and more restorations in children and adolescents with NF1 compared to controls, although based on panoramic radiographs [23]. Together, these findings highlight the influence of age, study design, and methodological differences on reported outcomes.

In our study, differences in caries experience between NF1 and matched controls were not observed in adolescents but were significant in adults. The absence of a difference in adolescents should be interpreted with caution due to the small number of matched pairs and wide confidence intervals, indicating substantial uncertainty. Thus, it remains unclear whether caries experience is truly similar in younger individuals with NF1 or whether a difference was not detected due to limited statistical power.

In contrast, the marked difference in DMFT observed in adults between NF1 and matched controls, together with the positive correlation between age and DMFT within the NF1 group, supports the hypothesis that increased caries experience in NF1 reflects a cumulative process that becomes more evident over time. This pattern is consistent with previous observations in the literature.

Although NF1 status was significantly associated with higher DMFT compared to matched controls, this difference was less evident when participants were categorized by caries severity. A higher proportion of individuals with NF1 showed high caries experience compared to matched controls, but this distribution was not statistically significant. This discrepancy likely reflects limited statistical power, as indicated by a post hoc power of 61.7%. Thus, the lack of significance in analysis should be interpreted with caution and may represent a Type II error rather than true clinical similarity between groups.

In our study, individuals with NF1 showed reduced salivary flow, increased viscosity, and a higher tongue coating index compared to matched controls. Most socioeconomic variables did not differ between groups; notably, the only significant difference was a lower proportion of renters in the NF1 group. These findings suggest that the higher caries experience in NF1 is unlikely to be explained by socioeconomic conditions. Consistently, housing status was not associated with DMFT within the NF1 group.

No significant association was observed between salivary flow parameters and DMFT in the NF1 group in bivariate analyses. However, the post hoc power for the UWSFR–DMFT correlation was low (20.4%), indicating limited ability to detect associations. Thus, although hyposalivation is prevalent in NF1, its contribution to caries experience may require a larger sample to be detected. Accordingly, within-group analyses should be interpreted as exploratory. Given the sample size and multiple comparisons, there is a risk of both Type I and Type II errors.

Furthermore, the distinct temporal nature of the variables should be considered: DMFT is a cumulative index reflecting lifetime disease [26, 27], whereas salivary flow and biochemical measures represent current physiological states. This mismatch is a known limitation of cross-sectional studies, as present salivary conditions may not directly reflect dental damage accumulated over time.

In contrast, the tongue coating index showed a significant positive correlation with DMFT in the NF1 group. This suggests that tongue coating reflects an oral environment with increased biofilm accumulation and poorer hygiene—factors associated with higher caries risk—rather than a direct causal factor. This interpretation is consistent with the ecological plaque hypothesis and current understanding of oral biofilm dynamics [37, 38].

The multivariable model confirmed that NF1 was independently associated with higher DMFT, even after adjustment for family income, oral hygiene (OHI-S), and dietary habits. Although income and oral hygiene were also significant, they did not fully explain the higher DMFT in the NF1 group. A significant interaction between group and cariogenic diet indicated that the association between diet and DMFT differed by group. In matched controls, lower frequency of cariogenic diet was associated with reduced DMFT, whereas this effect was attenuated in the NF1 group. These findings suggest that the association between diet and DMFT differs between groups and that individuals with NF1 may have increased susceptibility to caries, potentially attenuating the benefits of dietary improvements. This supports a multifactorial process in NF1, possibly involving intrinsic biological or structural oral factors beyond conventional behavioral and socioeconomic determinants, although such mechanisms cannot be inferred from the present model.

In addition to sample size–related limitations, other methodological aspects should be considered. The absence of interproximal radiographs, with diagnosis based on visual-tactile WHO criteria, may have underestimated the “decayed” component of DMFT in adults by failing to detect incipient or interproximal lesions. However, as examinations were performed by a single experienced examiner using a standardized approach across groups, this limitation is unlikely to have introduced differential bias, preserving the validity of intergroup comparisons.

Furthermore, the use of a convenience sample from a single reference center and the lack of random sampling may limit the external validity of our findings. However, internal validity was strengthened by the matched design and overall socioeconomic comparability between groups, with the NF1 group showing a lower proportion of renters than matched controls. Multivariable adjustment further reduced measured confounding, supporting that NF1 remained significantly associated with DMFT independent of these factors, although residual confounding cannot be excluded. Additionally, the absence of microbiological characterization may have restricted a more comprehensive understanding of the biological mechanisms underlying the observed associations.

Additionally, the assessment of salivary viscosity by visual thread formation presents a limitation due to its subjectivity and lack of objective quantification, although it is widely used as a clinical screening tool. This limitation was minimized by using a single experienced examiner; however, findings should be interpreted as indicative of altered salivary properties rather than a formal rheological characterization. Future studies using objective methods, such as viscometry, are needed to quantify these alterations and clarify the role of salivary rheology in oral health in NF1.

Although a validated food frequency questionnaire was used to assess cariogenic diet based on weekly consumption of sugar-containing foods and beverages [34, 35], this approach may not capture the full complexity of dietary exposure, including total fermentable carbohydrate load, intake of refined starch, and consumption patterns. Furthermore, dietary habits were assessed over a short-term period (7 days), whereas DMFT reflects cumulative lifetime caries experience, potentially leading to temporal mismatch and non-differential misclassification of exposure.

In this context, interpretation of cumulative caries experience requires caution due to the cross-sectional design. The lack of longitudinal dental records limited the identification of the primary cause of tooth loss. In adults, the “missing” component is less specific, as it may reflect causes other than caries, such as periodontal disease. However, the higher DMFT in NF1 was not restricted to a single component; increased scores across “decayed,” “missing,” and “filled” were observed compared to matched controls. This pattern suggests that higher DMFT in NF1 reflects a greater cumulative lifetime experience of dental caries.

Further investigation of microstructural and chemical properties of teeth in NF1—using techniques such as scanning electron microscopy (SEM), energy-dispersive X-ray spectroscopy (EDX), and micro-computed tomography (micro-CT)—is warranted to explore potential intrinsic defects in enamel and dentin mineralization. These aspects, along with the role of specific microbiological profiles, may help explain the increased caries experience in this population.

Future studies should include larger samples and move beyond cross-sectional designs; longitudinal approaches are needed to clarify causal relationships between salivary alterations and caries progression. In addition, molecular studies investigating *NF1* gene expression during odontogenesis may further elucidate the role of neurofibromin in tooth development.

## Conclusion

Individuals with NF1 present significantly higher DMFT compared to matched controls. Our findings suggest that this is a multifactorial process, characterized by an independent association between NF1 status and DMFT, even after adjusting for salivary flow, oral hygiene, socioeconomic status, and dietary habits. Notably, the high prevalence of hyposalivation and altered salivary viscosity—combined with the significant positive correlation between tongue coating and DMFT—highlight a complex and compromised oral environment in NF1.

## Data Availability

The datasets used and/or analyzed during the current study are available from the corresponding author on reasonable request.

## References

[1] Lee T-S, Chopra M, Kim RH et al (2023) Incidence and prevalence of neurofibromatosis type 1 and 2: a systematic review and meta-analysis. Orphanet J Rare Dis 18:292. 10.1186/s13023-023-02911-2 37710322 PMC10500831

[2] Tong J, Hannan F, Zhu Y et al (2002) Neurofibromin regulates G protein–stimulated adenylyl cyclase activity. Nat Neurosci 5:95–96 11788835 10.1038/nn792

[3] Gutmann DH, Collins FS (1993) The neurofibromatosis type 1 gene and its protein product, neurofibromin. Neuron 10:335–343 8461130 10.1016/0896-6273(93)90324-k

[4] Bergoug M, Doudeau M, Godin F et al (2020) Neurofibromin structure, functions and regulation. Cells 9:2365. 10.3390/cells9112365 33121128 PMC7692384

[5] Legius E, Messiaen L, Wolkenstein P et al (2021) Revised diagnostic criteria for neurofibromatosis type 1 and Legius syndrome: an international consensus recommendation. Genet Med 23:1506–151. 10.1038/s41436-021-01170-5 34012067 PMC8354850

[6] Jouhilahti E-M, Visnapuu V, Soukka T et al (2012) Oral soft tissue alterations in patients with neurofibromatosis. Clin Oral Investig 16:551–558. 10.1007/s00784-011-0519-x 21301902

[7] Shapiro SD, Abramovitch K, Van Dis ML et al (1984) Neurofibromatosis: oral and radiographic manifestations. Oral Surg Oral Med Oral Pathol 58:493–498 6436765 10.1016/0030-4220(84)90350-5

[8] D’Ambrosio, Langlais RP, Young RS (1988) Jaw and skull changes in neurofibromatosis. Oral Surg Oral Med Oral Pathol 66:391–396 3140162 10.1016/0030-4220(88)90252-6

[9] Kleinsorgen F, Luna EB, de Pinho Montovani P et al Fungiform Papillae and Gustatory Function in Neurofibromatosis Type 1: A Case–Control Study. Oral Diseases 31:656–671. 10.1111/odi.15148 39402886

[10] Tartaglia M, Gelb BD (2010) Disorders of dysregulated signal traffic through the RAS-MAPK pathway: phenotypic spectrum and molecular mechanisms. Ann N Y Acad Sci 1214:99–121. 10.1111/j.1749-6632.2010.05790.x 20958325 PMC3010252

[11] Chibly AM, Aure MH, Patel VN, Hoffman MP (2022) Salivary gland function, development, and regeneration. Physiol Rev 102:1495–1552. 10.1152/physrev.00015.2021 35343828 PMC9126227

[12] Wang B-L, Wang Z, Nan X et al (2019) Downregulation of microRNA-143-5p is required for the promotion of odontoblasts differentiation of human dental pulp stem cells through the activation of the mitogen-activated protein kinases 14-dependent p38 mitogen-activated protein kinases signaling pathway. J Cell Physiol 234:4840–4850. 10.1002/jcp.27282 30362514

[13] Goodwin AF, Tidyman WE, Jheon AH et al (2014) Abnormal Ras signaling in Costello syndrome (CS) negatively regulates enamel formation. Hum Mol Genet 23:682–692. 10.1093/hmg/ddt455 24057668 PMC3888259

[14] Luna EB, Montovani PP, Rozza-de-Menezes RE, Cunha KS (2021) Neurofibromin expression by normal salivary glands. Head Face Med 17:5. 10.1186/s13005-021-00256-4 33602260 PMC7890980

[15] Cunha K, Rozza-de-Menezes R, Luna E et al (2015) High prevalence of hyposalivation in individuals with neurofibromatosis 1: a case-control study. Orphanet J Rare Dis 10:24. 10.1186/s13023-015-0239-4 25759173 PMC4351927

[16] Pedersen AML, Sørensen CE, Proctor GB et al (2018) Salivary secretion in health and disease. J Oral Rehabil 45:730–746. 10.1111/joor.12664 29878444

[17] Van Nieuw Amerongen A, Bolscher JGM, Veerman ECI (2004) Salivary proteins: protective and diagnostic value in cariology? Caries Res 38:247–253. 10.1159/000077762 15153696

[18] Sejdini M, Meqa K, Berisha N et al (2018) The effect of Ca and Mg concentrations and quantity and their correlation with caries intensity in school-age children. Int J Dent 2018:2759040. 10.1155/2018/2759040 29853893 PMC5964478

[19] Lahiri D, Nag M, Banerjee R et al (2021) Amylases: biofilm inducer or biofilm inhibitor? Front Cell Infect Microbiol 11:660048. 10.3389/fcimb.2021.660048 33987107 PMC8112260

[20] Tucker T, Birch P, Savoy DM, Friedman JM (2007) Increased dental caries in people with neurofibromatosis 1. Clin Genet 72:524–527. 10.1111/j.1399-0004.2007.00886.x 18001351

[21] Visnapuu V, Pienihäkkinen K, Peltonen S et al (2011) Neurofibromatosis 1 and dental caries. Clin Oral Investig 15:119–121. 10.1007/s00784-009-0341-x 19727860

[22] Friedrich RE, Reul A (2018) Decayed, missing, and restored teeth in patients with Neurofibromatosis Type 1. J Clin Exp Dent 10:e107–e115. 10.4317/jced.54561 29670726 PMC5899786

[23] Friedrich RE, Schön M (2024) Dental Developmental Stages and Decayed, Missing, and Restored Teeth in Neurofibromatosis Type 1-affected Children and Adolescents. J Clin Exp Dent 16:e300–e322. 10.4317/jced.61363 38600934 PMC11003283

[24] Santoro R, Santoro C, Loffredo F et al (2020) Oral clinical manifestations of neurofibromatosis type 1 in children and adolescents. Appl Sci (Basel) 10:4687. 10.3390/app10144687

[25] Tsang ES, Birch P, Friedman JM et al (2010) Prevalence of dental caries in children with neurofibromatosis 1. Clin Oral Investig 14:479–480 author reply 480. 10.1007/s00784-009-0361-6 20049496

[26] Thota E, Veeravalli JJ, Manchala SK et al (2022) Age-dependent oral manifestations of neurofibromatosis type 1: a case–control study. Orphanet J Rare Dis 17:93. 10.1186/s13023-022-02223-x 35236379 PMC8889631

[27] Luna EB, Vieira GS, Motta FLK et al (2026) Periodontal disease and salivary gland dysfunction in neurofibromatosis type 1: A case–control study. Oral Dis. In press 10.1111/odi.7030641940749

[28] Petersen PE (2003) The World Oral Health Report 2003: continuous improvement of oral health in the 21st century – the approach of the WHO Global Oral Health Programme. Commun Dent Oral Epidemiol 31:3–24. 10.1046/j.2003.com122.x 15015736

[29] World Health Organization (2013) WHO | Oral health surveys: basic methods. WHO Press, World Health Organization, Geneva, Switzerland

[30] Carvalho PM, Castelo PM, Carpenter GH, Gavião MBD (2016) Masticatory function, taste, and salivary flow in young healthy adults. J Oral Sci 58:391–399. 10.2334/josnusd.16-0135 27665979

[31] Thyilstrup A, Fejerskov O (1995) Ecologia oral e cárie dentária. In: Cariologia clínica., 2^a^ Edition. Santos, SP, Brazil, pp 49–67

[32] Greene JC, Vermillion JR (1964) THE SIMPLIFIED ORAL HYGIENE INDEX. J Am Dent Assoc 68:7–13 14076341 10.14219/jada.archive.1964.0034

[33] Shimizu T, Ueda T, Sakurai K (2007) New method for evaluation of tongue-coating status. J Oral Rehabil 34:442–447. 10.1111/j.1365-2842.2007.01733.x 17518979

[34] Slater B, Philippi ST, Fisberg RM, Latorre MRDO (2003) Validation of a semi-quantitative adolescent food frequency questionnaire applied at a public school in São Paulo, Brazil. Eur J Clin Nutr 57:629–635. 10.1038/sj.ejcn.1601588 12771963

[35] Moimaz SAS, Zina LG, Serra FAP et al (2011) Analysis of the Diet and Oral Health Condition in Pregnant Patients. Brazilian Res Pediatr Dentistry Integr Clin 10:357–363

[36] Ministério da Saúde SB (2011) Brasil 2010: Pesquisa Nacional de Saúde Bucal-Resultados Principais. Ministério da Saúde, Brasília

[37] Mosaddad SA, Tahmasebi E, Yazdanian A et al (2019) Oral microbial biofilms: an update. Eur J Clin Microbiol Infect Dis 38:2005–2019. 10.1007/s10096-019-03641-9 31372904

[38] Marsh PD (2018) In Sickness and in Health-What Does the Oral Microbiome Mean to Us? An Ecological Perspective. Adv Dent Res 29:60–65. 10.1177/0022034517735295 29355410

